# Impact of the COVID-19 lockdown period on adult musculoskeletal injuries and surgical management: a retrospective monocentric study

**DOI:** 10.1038/s41598-020-80309-x

**Published:** 2020-12-31

**Authors:** V. Crenn, M. El Kinani, G. Pietu, M. Leteve, M. Persigant, C. Toanen, Y. Varenne, N. Goffinet, K. Buffenoir, F. Javaudin, E. Montassier

**Affiliations:** 1 Orthopedics and Trauma Department, University Hospital Hôtel-Dieu, UHC of Nantes, 1 Place Alexis Ricordeau, 44093 Nantes Cedex 1, France; 2 PhyOs 1238, INSERM, University of Nantes, Nantes, France; 3 Emergency Department, University Hospital Hôtel-Dieu, UHC of Nantes, Nantes, France; 4 Neurosurgery Department, University Hospital Hôtel-Dieu, UHC of Nantes, Nantes, France

**Keywords:** Medical research, Epidemiology, Fracture repair, Musculoskeletal system, Orthopaedics, SARS-CoV-2

## Abstract

The coronavirus disease 2019 (COVID-19) pandemic has led to the worldwide implementation of unprecedented public protection measures. On the 17th of March, the French government announced a lockdown of the population for 8 weeks. This monocentric study assessed the impact of this lockdown on the musculoskeletal injuries treated at the emergency department as well as the surgical indications. We carried out a retrospective study in the Emergency Department and the Surgery Department of Nantes University Hospital from 18 February to 11 May 2020. We collected data pertaining to the demographics, the mechanism, the type, the severity, and inter-hospital transfer for musculoskeletal injuries from our institution. We compared the 4-week pre-lockdown period and the 8-week lockdown period divided into two 4-week periods: early lockdown and late lockdown. There was a 52.1% decrease in musculoskeletal injuries among patients presenting to the Emergency Department between the pre-lockdown and the lockdown period (weekly incidence: 415.3 ± 44.2 vs. 198.5 ± 46.0, respectively, p < .001). The number of patients with surgical indications decreased by 33.4% (weekly incidence: 44.3 ± 3.8 vs. 28.5 ± 10.2, p = .048). The policy for inter-hospital transfers to private entities resulted in 64 transfers (29.4%) during the lockdown period. There was an increase in the incidence of surgical high severity trauma (Injury Severity Score > 16) between the pre-lockdown and the early lockdown period (2 (1.1%) vs. 7 (7.2%), respectively, p = .010) as well as between the pre-lockdown and the late lockdown period (2 (1.1%) vs. 10 (8.3%), respectively, p = .004). We observed a significant increase in the weekly emergency department patient admissions between the early and the late lockdown period (161.5 ± 22.9, 235.5 ± 27.7, respectively, p = .028). A pronounced decrease in the incidence of musculoskeletal injuries was observed secondary to the lockdown measures, with emergency department patient admissions being halved and surgical indications being reduced by a third. The increase in musculoskeletal injuries during the late confinement period and the higher incidence of severe trauma highlights the importance of maintaining a functional trauma center organization with an inter-hospital transfer policy in case of a COVID-19s wave lockdown.

## Introduction

The coronavirus disease 2019 (COVID-19) pandemic has led to the worldwide implementation of unprecedented public protection measures. France reached the first 10 cases milestone on the 18th of February, and since the 29th of February, several stages of protection measures have been implemented (prohibition of public gatherings, school closures, and incentives for working remotely). The population lockdown was announced on the 17th of March 2020 for 8 weeks until the 11th of May 2020. The hospital workload from patients in the emergency department (ED) can vary during exceptional events of various natures in Europe (terrorist attacks^1,2^) and the world (natural disasters^3,4^). While there is precedent for large disasters of natural or human origin in recent history, such as pandemics like H1N1^5^, none have been as long-lasting or as global as the COVID-19 pandemic.

The COVID-19 lockdown is leading to limitations being placed on work, sports, leisure activities, road traffic circulation, as well as in regard to surgery. All elective surgeries except oncological or septic non-delayable procedures have had to be rescheduled. Despite a pronounced decline in the influx of patients with musculoskeletal injuries (MSI), trauma activity remains a necessity, from reception in the emergency department to treatment in the operating room^6,7^. Nantes University Hospital is the primary referral center for major trauma, neurosurgery, and orthopaedics, with more than 125,000 emergency admissions and more than 45,000 surgeries per year in 2018 and a catchment area of more than 3.5 million people. Nantes University Hospital is recognized as one of the leading French trauma centers, providing treatment to high severity trauma patients who require multidisciplinary care, and it remains representative in terms of the MSI influx and management. Our surgical technical platform comprises 22 operating theaters (including six for orthopaedics and neurosurgery, and three emergency operating rooms shared among other specialties). During the lockdown, this capacity was reduced to six rooms, including four emergency operating rooms shared between the various specialties. All non-emergency surgical procedures were canceled, rescheduled, or transferred. Inter-hospital transfers from our institution to private hospitals were set up on the 17th of March to the only three private medical facilities in Nantes with orthopedic surgeons and neurosurgeons.

There has been little quantitative or qualitative data to date regarding the impact of lockdown measures on emergency departments with a focus on surgical traumatology^8,9^. Our goal was to assess the impact of the COVID-19 lockdown on the hospital workload from patients presenting to the emergency department and the operating room for musculoskeletal injuries since the beginning of containment measures. We compared data for the pre-lockdown (PL) and the lockdown (L) period, divided into early (L1) and late lockdown (L2), with data from previous years (2018 and 2019). We analysed the type of injuries, the severity, the mechanism, and the rate of surgical indications.

## Materials and methods

Data on ED admissions at Nantes University Hospital between 18 February (the 10th case milestone of COVID-19 in France) and 11 May 2020 were collected from centralized hospital registries. These data were collected prospectively from the Emergency Department and the Orthopaedics and Neurosurgery Department registries and analysed retrospectively. Each patient admitted for a musculoskeletal injury (defined by the ICD-10 classification (see Supplementary Table 1, Additional File 1) and cross-checked with injury admissions patterns) in the adult emergency department were included. The first dataset from the emergency department registry contained socio-demographic information and admissions data. The second dataset from the Orthopaedics and Neurosurgery departments collected the surgical indications in limbs, and the axial musculoskeletal injuries operated on at Nantes University Hospital, or records of an inter-hospital transfer from our institution to another facility. The patient datasets underwent manual verification in the original patient files in case of missing data or contradictory information. In the emergency department admissions for the musculoskeletal injuries dataset, we collected the gender, age, and date of admission. In the dataset for surgical indications, we completed the data collection with transfer events, the date of surgery, the injury localization (lower or upper limb, spine, and poly-fractured), the severity (defined as an Injury Severity Score (ISS) > 16^10,11^), the mechanism (work accident, domestic accident, road accident, assault, suicide attempt, sport accident, elderly fall^12^), and drug use.

The primary objective was to compare the number of emergency department admissions and surgical procedures between the pre-lockdown and the lockdown period. The secondary objectives were: (a) to analyse the qualitative and quantitative data on surgical indications between the pre-lockdown period and between the early period (L1) and the late lockdown (L2) period, and to analyse the transfer rate. (b) We also compared the quantitative data to the 2018 and 2019 reference years from 03/17 to 05/11 during the same lockdown period. Moreover, we divided the lockdown period into two equal shorter time intervals to increase the precision and to capture subtle particularities due to the end of the lockdown.

We classified different periods in 2020:

1. Pre-lockdown (PL): a 4-week period from 18 February to 16 March 2020.
2. Lockdown (L): an 8-week period from 17 March to 11 May 2020. This period was split into two equal 4-week periods with similar lockdown restrictions and protection measures:
  - Early lockdown (L1): 4 weeks from 17 March to 13 April 2020,
  - Late lockdown (L2): 4 weeks from 14 April to 11 May 2020.

The institutional review board approved the protocol, and all documents and procedures were reviewed and approved by the NGEHS (*Nantes Group for Ethics in the Health Sector)* Ethics Committee (30th of April 2020), due to anonymized data on retrospective study, the NGEHS Ethics Committee has approved to waive patient consent. Methods were carried out in accordance with relevant guidelines and regulations.

The data are presented as means with the standard deviation (except for age, for which the median is indicated), or as absolute values associated with the percentage. The qualitative and ordinal variables were compared using the Chi^2^ test (Fisher’s exact test was performed when Chi^2^ validity conditions were not met). The quantitative variables were analysed with a t-test or a one-way ANOVA test (with Levene’s test for variance homogeneity assumption and Tukey’s test for post hoc analysis). Non-parametric Wilcoxon–Mann–Whitney or Kruskal–Wallis tests were performed when parametric conditions were not met. We used Pearson’s correlation coefficient to investigate the relationship between two quantitative variables. The alpha risk of all these tests was set at 5%, with a level of significance of p < 0.05. The data collection was performed with Microsoft Excel, and the statistical analyses were performed using IBM SPSS Statistics V25 software.

### Ethics approval and consent to participate

The institutional review board approved the protocol, and all documents and procedures were reviewed and approved by the NGEHS (*Nantes Group for Ethics in the Health Sector)* Ethics Committee (30th of April 2020), due to anonymized data on retrospective study, the NGEHS Ethics Committee has approved to waive patient consent. Methods were carried out in accordance with relevant guidelines and regulations.

## Results

### Pre-lockdown period and lockdown period

**Figure 1:**
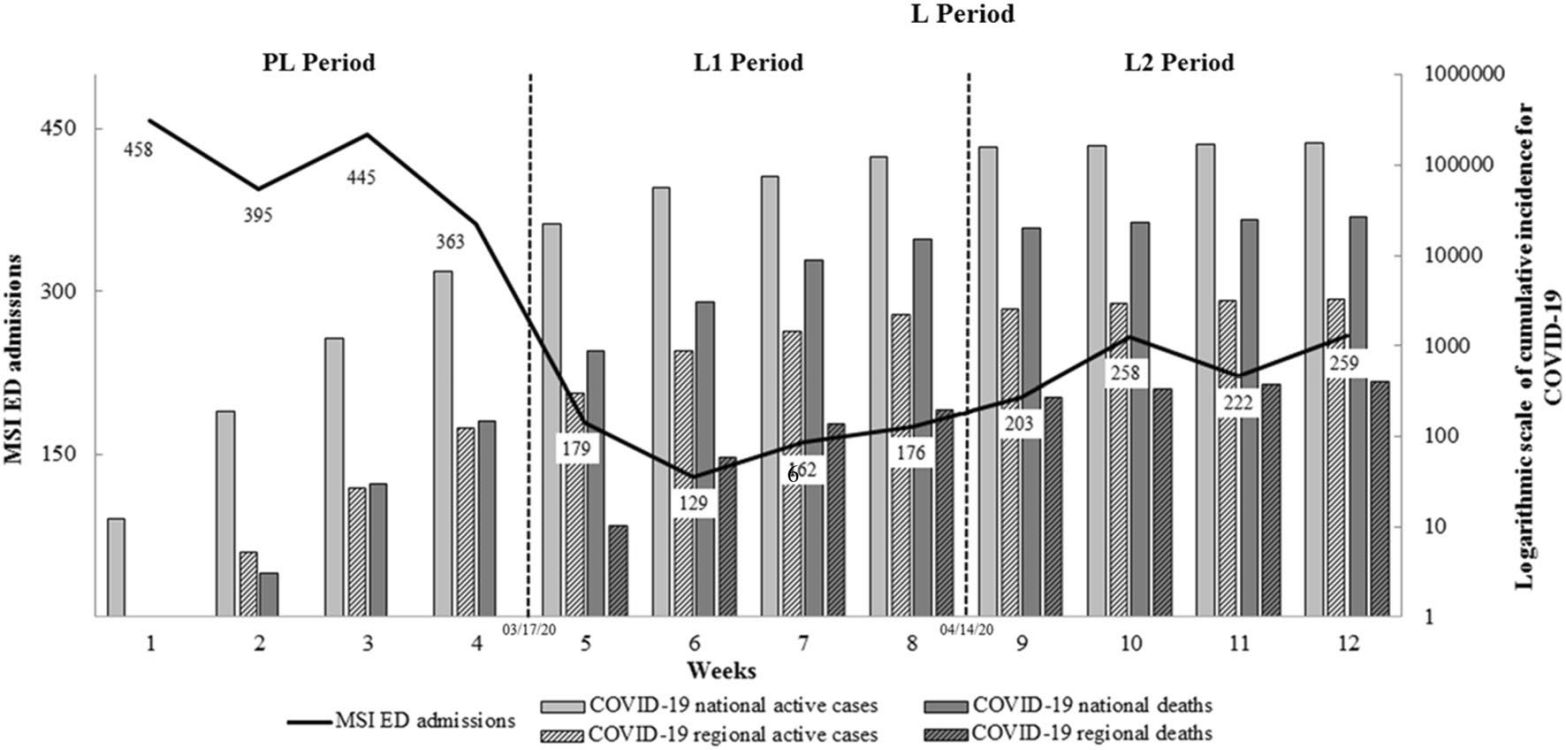
Change in the weekly admissions for MSI in the ED at Nantes University Hospital (linear scale graph), and the weekly cumulative incidence of COVID-19 national and regional active cases and deaths (logarithmic scale bar graphs) between the PL and L periods The first vertical line refers to the national French lockdown on the 17th of March 2020. The second vertical line refers to the virtual break separating L1 (Early lockdown period) and L2 (Late lockdown period) on the 14th of April 2020. MSI: Musculoskeletal injury, ED: Emergency department, PL: Pre-lockdown (from 02/18/20 to 03/16/20), L: Lockdown (from 03/17/20 to 05/11/20), L1: Early lockdown, L2: Late lockdown.

We found that there was a significant decrease of 52.1% in the weekly emergency department admissions for musculoskeletal injuries (415.3 ± 44.2 vs. 198.5 ± 46.0, p < 0.001) and of 33.4% for the weekly surgical procedures for musculoskeletal injuries (44.3 ± 3.8 vs. 28.5 ± 10.2, p < 0.001) between the pre-lockdown and the lockdown period, respectively (Fig. 1). There was likewise an increase in the surgical procedures ratio (180/1661 (10.8%) vs. 217/1588 (13.7%), p = 0.014).

In terms of emergency department admissions, there was a significant difference in age, with younger patients in the pre-lockdown period compared to the lockdown period (median age: 34.8 ± 21.2 vs. 43.0 ± 22.7, respectively, p < 0.001). There was no difference in the sex-ratio between these two periods (women: 689 (41.5%) vs. 641 (42.9%), respectively, p = 0.513).

For the surgical procedures, the Chi^2^ test showed a significantly higher proportion of women in the pre-lockdown period compared to the lockdown period (105 (58.3%) vs. 96 (44.2%), respectively, p = 0.005), while there was no difference in age (median age: 71.5 ± 20.8 vs. 66 ± 19.5, respectively, p = 0.352).

When we removed the first-week outlier from the lockdown period analysis, we obtained a significant and robust correlation (ρ = 0.982, p < 0.001) between the number of emergency department admissions and the surgical procedures (correlation including the first week: ρ = 0.390, p = 0.340).

**Figure 2:**
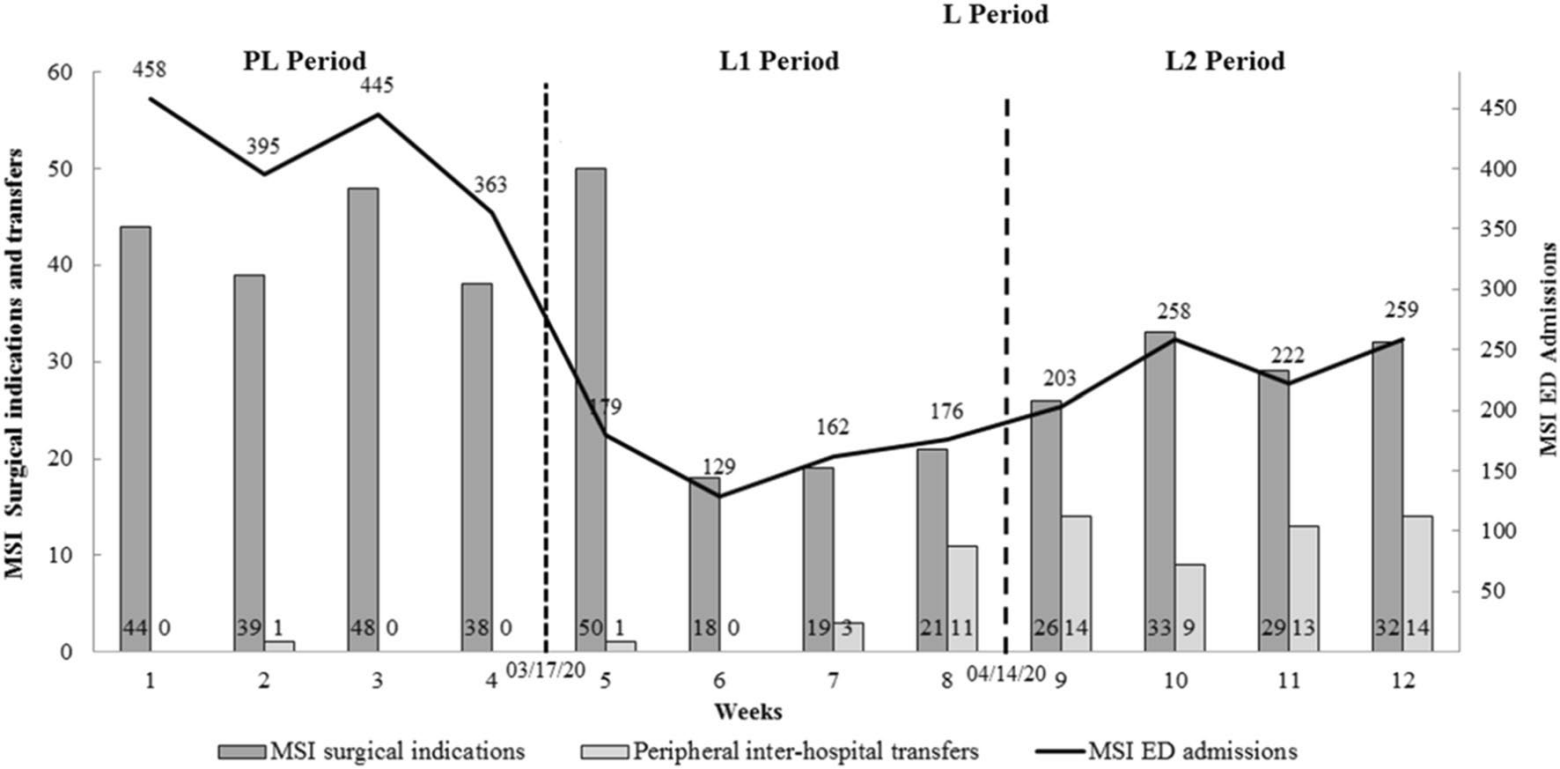
Change in the weekly surgical procedures, peripheral inter-hospital transfers, and weekly admissions for MSI in the ED of Nantes regional University Hospital (linear scale graph). The first vertical line refers to the national French lockdown on the 17th of March 2020. The second vertical line refers to the virtual break separating L1 (Early lockdown period) and L2 (Late lockdown period) on the 14th of April 2020. MSI: Musculoskeletal injury, ED: Emergency department, PL: Pre-lockdown (from 02/18/20 to 03/16/20), L: Lockdown (from 03/17/20 to 05/11/20), L1: Early lockdown (from 03/17/2020 to 04/13/20), L2: Late lockdown (from 04/14/20 to 05/11/20).

We found that there was a significant increase in the inter-hospital transfer rate between the pre-lockdown and the lockdown period for surgical procedures (1 (0.6%) vs. 64 (29.4%), respectively. p < 0.001) (Fig. 2).

### Lockdown period

When comparing the early (L1) and the late (L2) lockdown period, we noticed a significant increase of 45.5% in emergency department admissions for musculoskeletal injuries (23.1 ± 6.0 vs. 33.6 ± 6.5, respectively, p < 0.001) and a significant increase of 142.4% in the inter-hospital transfers rate (16 (16.5%) vs. 48(40%), respectively, p < 0.001).

An increase in domestic accidents between the early (L1) and the late (L2) lockdown period (22 (22.7%) vs. 33 (27.5%), respectively, p = 0.017) was also noted. We observed a significant decrease in the surgical indications in terms of the elderly fall mechanism between the early (L1) and the late (L2) lockdown period (55 (56.7%) vs. 47 (39.2%), respectively, p = 0.010). However, an increase in surgical indications for road accidents (6 (6.2%) vs. 17 (14.2%), p = 0.057), sports accidents (1 (1.0%) vs. 7 (5.8%), p = 0.078), and suicide attempts (1 (1.0%) vs. 3 (2.5%), p = 0.630) was found between the L1 and the L2 lockdown period, without it being significant (Table 1).

**Table 1:** Patient characteristics for MSI in ED admissions and MSI surgical indications.Bold values denote statistical significance at the p < 0.05 level.

	Pre-lockdown period	Lockdown period		p-value		
		L1	L2			
	28 days from 02/18 to 03/16	28 days from 03/17 to 04/13	28 days from 04/14 to 05/11	PL vs. L1	PL vs. L2	L1 vs. L2
**ED admission data**	n = 1661	n = 646	n = 942			
MSI daily patients^a^	59.3 ± 10.1	23.1 ± 6.0	33.6 ± 6.5	** < .001**	** < .001**	**.002**
MSI ED patients/week	415.3 ± 44.2	161.5 ± 22.9	235.5 ± 27.7	** < .001**	** < .001**	**.028**
Median age, years^a^	34.8 ± 21.2	44.2 ± 23.3	43.1 ± 22.3	** < .001**	** < .001**	1
Women	689 (41.5%)	255 (39.5%)	386 (41.0%)	.379	.801	.551
**Surgical indication data**	n = 180	n = 97	n = 120			
MSI surgical indications ratio	180/1661 (10.8%)	97/646 (15.0%)	120/942 (12.7%)	**.006**	.144	.194
MSI daily surgical indications	6.3 ± 2.2	3.5 ± 2.5	4.3 ± 1.8	** < .001**	** < .001**	.757
MSI surgical indications/week	44.3 ± 3.8	24.2 ± 9.9	30.0 ± 3.2	**.003**	**.018**	.417
Transfers/week	1 (0.6%)	16 (16.5%)	48 (40%)	** < .001**	** < .001**	** < .001**
Median age, years	71.5 ± 20.8	73 ± 22.5	60 ± 22.3	.736	.125	**.047**
Women	105 (58.3%)	45 (46.4%)	51 (42.5%)	.057	**.007**	.566
**Mechanism**						
Elderly fall	99 (55%)	55 (56.7%)	47 (39.2%)	.786	**.007**	**.010**
Road accident	20 (11.1%)	6 (6.2%)	17 (14.2%)	.180	.106	.057
Domestic accident	29 (16.1%)	22 (22.7%)	33 (27.5%)	.178	**.017**	.417
Sport accident^b^	9 (5%)	1 (1.0%)	7 (5.8%)	.172	.797	.078
Work accident^b^	10 (5.6%)	5 (5.2%)	4 (3.3%)	.888	.371	.518
Assault^b^	7 (3.9%)	5 (5.2%)	6 (5.0%)	.758	.774	1
Suicide attempt^b^	1 (0.6%)	1 (1.0%)	3 (2.5%)	1	.306	.630
Others^b^	5 (2.8%)	2 (2.1%)	3 (2.5%)	1	1	1
**Localization**						
Upper limb	37 (20.6%)	14 (14.4%)	28 (23.3%)	.210	.567	.099
Lower limb	105 (58.3%)	55 (56.7%)	57 (47.5%)	.793	.065	.177
Spine	23 (12.8%)	13 (13.4%)	20 (16.7%)	.883	.346	.505
Poly-fractured	15 (8.3%)	15 (15.5%)	15 (12.5%)	.068	.239	.529
**High severity trauma**						
ISS > 16^b^	2 (1.1%)	7 (7.2%)	10 (8.3%)	**.010**	**.004**	.805
Drug use	15 (8.3%)	6 (6.2%)	10 (8.3%)	.519	1	.547

Chi^2^ square test and one-way ANOVA test;

ED: Emergency department; L: Lockdown; PL: Pre-lockdown; L1: Early lockdown; L2: Late lockdown; MSI: Musculoskeletal indications; ISS: Injury Severity Score.

^a^Kruskal-Wallis non-parametric-test.

^b^Fischer’s exact test.

**Figure 3:**
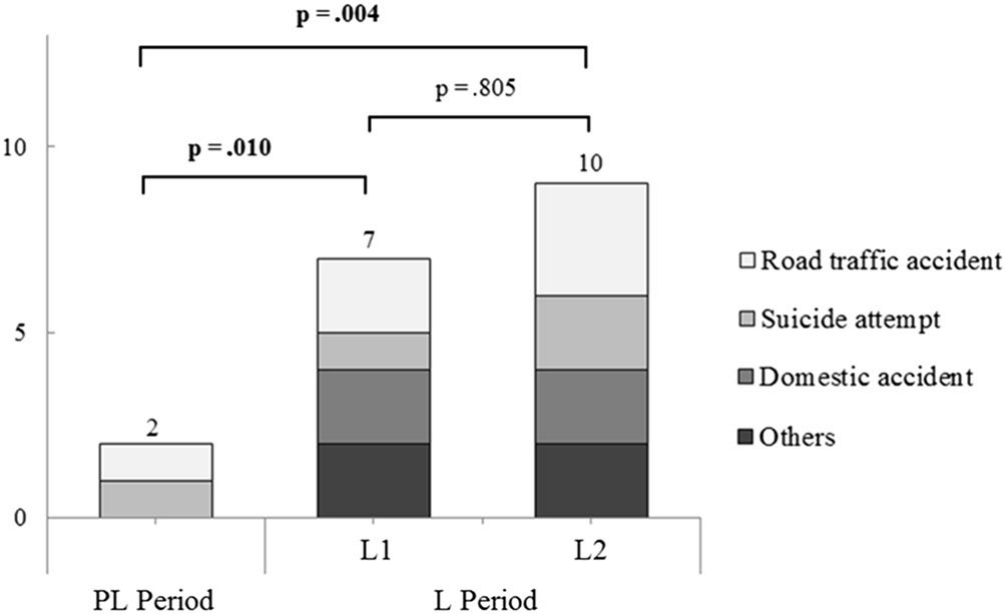
Stacked bar graph representation of high severity trauma (ISS > 16) according to the mechanism and the PL, L1, and L2 periods, with significant differences in the Fisher’s exact test. Others: L1: one assault, one work accident; L2: one sports accident, one work accident. PL: Pre-lockdown (from 02/18/20 to 03/16/20), L: Lockdown (from 03/17/20 to 05/11/20), L1: Early lockdown (from 03/17/2020 to 04/14/2020), L2: Late lockdown (from 04/14/2020 to 05/11/2020); ISS: International Severity Score.

We observed a significant increase in high severity trauma (ISS > 16) patients between the pre-lockdown (2 (1.1%)) and the early lockdown period (7 (7.2%), p = 0.010), as well as between the pre-lockdown (2 (1.1%)) and the late lockdown (10 (8.3%), p = 0.002) (Fig. 3).

Our analysis revealed a significant decrease in emergency department admissions between 2020 (n = 1588) and 2018 (n = 4188) and 2019 (n = 4414) of 62.1% and 64.0%, respectively. The patients were significantly older in the 2020 lockdown period (2018: 33.7 ± 20.6, 2019: 34.0 ± 20.9, 2020: 43.0 ± 22.7, p < 0.001 for both comparisons). The surgical indications ratio was significantly higher in 2020 (217/1588, 13.7%) compared to 2018 (339/4188, 8.1%) (p < 0.001) and 2019 (354/4414, 8.0%) (p < 0.001). No significant differences between 2018 and 2019 were identified for any of the criteria (see Supplementary Figure 1, Additional File 2, and Supplementary Table 2, Additional File 3).

## Discussion

The worldwide pandemic and containment strategies due to COVID-19 are unprecedented. Influenza pandemic health strategies and behavioral modifications in Asia (with H1N1) have already been studied^5,13^, but they are not comparable to the sudden COVID-19 containment measures in Europe. Lockdown appears to be able to control COVID-19, while also reducing the occurrence of musculoskeletal injuries. The need for trauma service reorganization from emergency to surgery departments was nonetheless necessary^7,14^.

A pronounced 52.1% decrease in weekly emergency department admissions between the pre-lockdown (415.3 ± 44.2) and the lockdown period (198.5 ± 46.0) was noted in our hospital (p < 0.001). The magnitude of this decrease is greater than the 36.9% decrease described by Hampton et al. for a shorter 2-week period before and after a lockdown in Britain^8^. Compared to 2018 and 2019, the decrease in emergency department musculoskeletal injuries admissions in the lockdown period in 2020 was decreased by 62.1 and 64%, respectively. This drop in admissions allowed for a supervised reorganization of our para-medical staff to better respond to the influx of COVID-19 cases. Thus, an emergency doctor was able to be mobilized to assess patients suspected of having COVID-19. Trauma admission care was achieved with a limited number of beds and staffed by a team of two emergency residents and a senior orthopaedic surgeon. On the other hand, the pronounced decrease in emergency department admissions raised the question of delayed diagnosis and self-medication hypothesis for some patients, as described by Italian medical teams^15,16^. Musculoskeletal sequelae or chronic pathologies resulting from a fear of contracting COVID-19 may be seen in the months following the lockdown.

The decrease in emergency department admissions was linked to a reduction in surgical indications for musculoskeletal injuries of 33.4% between the pre-lockdown and the lockdown period (36.1% between 2018 and 2020, and 38.1% between 2019 and 2020). Park et al. observed a similar decrease in their study of the trauma workload in England^9^. Conversely, the surgical indications rate increased from 10.8% in the pre-lockdown period to 15.0% (p = 0.006) in the early lockdown period (L1), followed by a decrease of 12.7% in the late lockdown period (L2). The overall lockdown rate of surgical procedures for emergency admissions was significantly higher (13.7%) compared to 2018 (8.1%) and 2019 (8.0%), illustrating the fact that patients consulted less for minor injuries during the lockdown.

We reported an increase in inter-hospital transfers from our hospital to private surgical facilities, going from 1 (0.6%) in pre-lockdown to 64 (29.4%) in the lockdown period (p < 0.001). The increment between the early lockdown and the late lockdown period was also significant, going from 16 (16.5%) to 48 (40%) (p < 0.001), which demonstrates the improvement in inter-hospital transfers organization. General elected surgery rescheduling affected private structures during the whole lockdown period, except for emergency procedures. Thus, their surgical teams were available and in financial need for the trauma surgery transfers that were allowed. These transfers were essential as the operational capacity of our operating theaters was reduced due to the allocation of respirators, medical and para-medical staff for COVID-19-related treatments. The inter-hospital transfer policy to private institutions was a necessity and allowed us to maintain quality management for emergency surgeries. We used a management algorithm similar to those reported elsewhere in Europe^6,7^. In the event of a surgical indication in a patient with mild or minor injury without symptoms of Coronavirus infection, transfer to a private hospital was systematically considered. COVID-19-positive patients, or patients who could not be transferred due to their health status, were treated at the Nantes regional University Hospital.

Surgical procedures were lower for all mechanisms, except for domestic injuries, which increased significantly between the pre-lockdown and the late lockdown period (29 (16.1%) vs. 33 (27.5%), p = 0.017). This has also been described by Hamptons et al. and seems logical due to confinement measures^8^; some of these injuries amounted to high severity trauma such as injuries sustained from a fall from a roof or a tree. The proportion of surgical indications for elderly fragility fractures remained equivalent for the pre-lockdown, early, and late lockdown periods, although the absolute numbers were halved compared to the pre-lockdown period. It seems possible that some of these older patients were at first redirected to other local facilities to avoiding the main medical facility where most COVID-19 patients were being treated. Limitation of activity, with isolation of the elderly, and containment measures in retirement homes could explain this phenomenon. Elderly patients are also known to comply better with containment measures due to a pandemic^5,13^.

In the last 4 weeks of the lockdown (L2) there was a 45.5% increase in weekly emergency department admissions compared to the early lockdown period (L1) (23.1 ± 6.0 vs. 33.6 ± 6.5, p < 0.001), there was also an increase in the weekly surgical procedures (24.2 ± 9.9 vs. 30.0 ± 3.2, p = 0.417). This may reflect the difficulty with maintaining strict confinement for a long time, with population behavioral fatigue related to these restrictive measures^17^. The improvement in the COVID-19 pandemic situation in France could also underlie this rebound in admissions, with a return to work for some activities. From a musculoskeletal injury point of view, the prolonged duration of confinement appeared to increase the occurrence of high severity trauma. The proportion of high severity trauma with an ISS > 16 requiring surgery increased drastically between the pre-lockdown (2 (1.1%)) and the early lockdown period (7 (7.8%), (p = 0.010)), with most of these being due to road traffic injuries, suicide attempts, and domestic accidents. This result was consistent with the late lockdown period (10 (8.3%))^18,19^. This phenomenon could be explained by behavioral changes due to confinement. Given the decrease in road traffic, one hypothesis is that unsafe driving behavior increased, thereby leading to more severe trauma. We also hypothesize that suicide attempts were more frequent in the late confinement period due to prolonged stress, isolation, and lack of psychiatric support^17,18^. Lastly, people spending time at home engaged in home improvements or do-it-yourself tasks that resulted in falls from ladders or roofs, as described by Park et al.^9^.

The observations made during this study may be transposable to other equivalent entities in terms of infrastructure and population base, although it should also be linked to the pandemic status of the region. Western France was the last area affected by COVID-19: the Pays de la Loire region was able to promptly implement the necessary lockdown measures. This geographic “benefit” probably improved the results for infection control, and it could also have impacted the number of individuals presenting with musculoskeletal injuries at our hospital.

During this study, all of the data were collected via the digital emergency files. Given the large workforce recorded during these periods, there are probably limitations regarding the accuracy of the incident and the reasons for the admissions of patients with musculoskeletal injuries. However, the same analysis procedure was carried out over the various pre-lockdown and lockdown periods, as well as in 2018 and 2019, which hence makes it possible to obtain comparable data. The criterion of the severity of the trauma, based on the assessment of the doctor in attendance, corresponds to an examination carried out in an emergency without subsequent re-evaluation, which can then either increase or decrease the lesions noted, particularly after surgical exploration. Seasonality may also have an impact on the number of admissions in 2019 versus 2020, due to weather conditions or social events such as the yellow jacket movement in 2019. Our analysis lacks qualitative data regarding the surgical procedures in 2018 and 2019, and a comparison with the pre-lockdown and lockdown periods, as well in 2018 and 2019. It seems possible that the pre-lockdown period studied in 2020 was already impacted by the spread of COVID-19 and early protection measures with activity limitations.

The availability of private entities with trauma resources was instrumental in avoiding overloading of our facility. However, even though 64 transfers (29.4%) were recorded during the lockdown period, we were unable to determine the share of activity linked to direct admissions to these external entities. Indeed, since these private facilities also provide emergency treatments, Nantes University Hospital is not the only direct reception center for musculoskeletal injuries. The assessment based on the Nantes regional University Hospital admissions may underestimate the overall number of musculoskeletal injuries, especially for older patients with fragility fractures. This is less so for high severity or spinal traumas, as the Nantes regional University Hospital is the referral center and there are not many teams capable of dealing with these types of trauma in private centers and in outlying areas.

## Conclusion

Overall, we observed a significant decrease in emergency admissions of trauma patients during the COVID-19 lockdown period. The containment measures, in addition to limiting the spread of the epidemic, also had a significant impact on the number of musculoskeletal injuries requiring surgical intervention. This approximately 50% reduction in emergency department admissions, and the decrease in surgical indications by a third, allowed reorganization of the emergency and the surgical department to better accommodate all patients in conjunction with the other smaller medical facilities. However, we observed an increase in the frequency of high severity trauma during the lockdown and an upsurge in musculoskeletal injuries in the late lockdown period, which probably reflects the behavioral fatigue stemming from prolonged restrictions. These results highlight the need for a strictly functional trauma center, as well as inter-hospital transfer solutions in case of a COVID-19s wave lockdown^20^.

## Supplementary Information

Supplementary Information.

## Data Availability

The data that support the findings of this study are available on request from the corresponding author.

## Abbreviations

COVID-19: Coronavirus disease 2019
ED: Emergency department
L: Lockdown
PL: Pre-lockdown
L1: Early lockdown
L2: Late lockdown
MSI: Musculoskeletal injury
NGEHS: Nantes Group for Ethics in the Health Sector
ISS: Injury Severity Score
