# Supplementary material for: Impact of the COVID-19 lockdown period on adult musculoskeletal injuries and surgical management: a retrospective monocentric study
Source: Sci Rep. 2020 Dec 31;10:22442. doi: 10.1038/s41598-020-80309-x (PMC7775434; doi:10.1038/s41598-020-80309-x)
Supplement: Supplementary file 1 — Supplementary Information. [file 41598_2020_80309_MOESM1_ESM.docx]

**Manuscript title:** *Impact of the Covid-19 lockdown period on adult musculoskeletal injuries and surgical management: a retrospective monocentric study*

**Running head title:** *COVID-19 lockdown and musculoskeletal injuries*

**V. Crenn^1,2^, M. El Kinani^1^, G. Pietu^1^, M. Leteve^1^, M. Persigant^1^, C. Toanen^1^, Y. Varenne^1^, N. Goffinet^3^, K. Buffenoir^4^, F. Javaudin^3^, E. Montassier^3^**

**Author details:**

1: Orthopedics and Trauma Department, University Hospital Hôtel-Dieu, UHC of Nantes, France

2: PhyOs 1238, INSERM, University of Nantes, France

3: Emergency Department, University Hospital Hôtel-Dieu, UHC of Nantes, France

4: Neurosurgery Department, University Hospital Hôtel-Dieu, UHC of Nantes, France

**Corresponding Author:**

Vincent CRENN, MD

Orthopedics and Trauma Department, University Hospital Hôtel-Dieu, UHC of Nantes, 1 place Alexis Ricordeau, 44093 Nantes cedex 1, France

E-mail: [vincent.crenn@chu-nantes.fr](mailto:vincent.crenn@chu-nantes.fr)

**Additional File 1:**

**Supplementary Table 1: ICD-10 classification included in the study:**

| **ICD-10 classification:** |
| --- |
| CHAPTER XIX |
| S12 Fractures of the neck  Excluding S12.8 Fractures of other parts of the neck  S13 Dislocation, sprain and strain of joints and ligaments in the neck  Excluding S13.5 Sprains and strains of the thyroid region  S14 Traumatic injuries to the nerves of the spinal cord in the neck  Excluding S14.4 Traumatic injuries to other nerves in the neck  S22 Traumatic lesions of the ribs, sternum, dorsal spine  Excluding S22.2 to S22.9  S23 Dislocation, sprain and strain of joints and ligaments in the chest  Excluding S23.4 and S23.5  S24 Traumatic injuries to the nerves of the spinal cord in the thorax  Excluding S24.3 to S24.6  S32 Lumbar spine and pelvis fracture  S33 Dislocation, sprain and strain of the joints and ligaments of the lumbar spine and pelvis  S34 Traumatic injury to the nerves and lumbar spinal cord in the abdomen, lower back and pelvis  S40-S49 Traumatic injuries to the shoulder and arm  S50-S59 Traumatic injuries to the elbow and forearm  S60-S69 Traumatic injuries to the wrist and hand  S70-S79 Traumatic injuries to the hip and thigh  S80-S89 Traumatic injuries to the knee and leg  S90-S99 Traumatic injuries to the ankle and foot  T00-T07 Traumatic injuries to several parts of the body  T08-T14 Traumatic lesions of unspecified part of the trunk |
| CHAPTER XX |
| V01-V99 Transport accidents  W00-W19 Falls  X60-X84 Self-inflicted injuries  X85-Y09 Assaults |

**Additional File 2:**


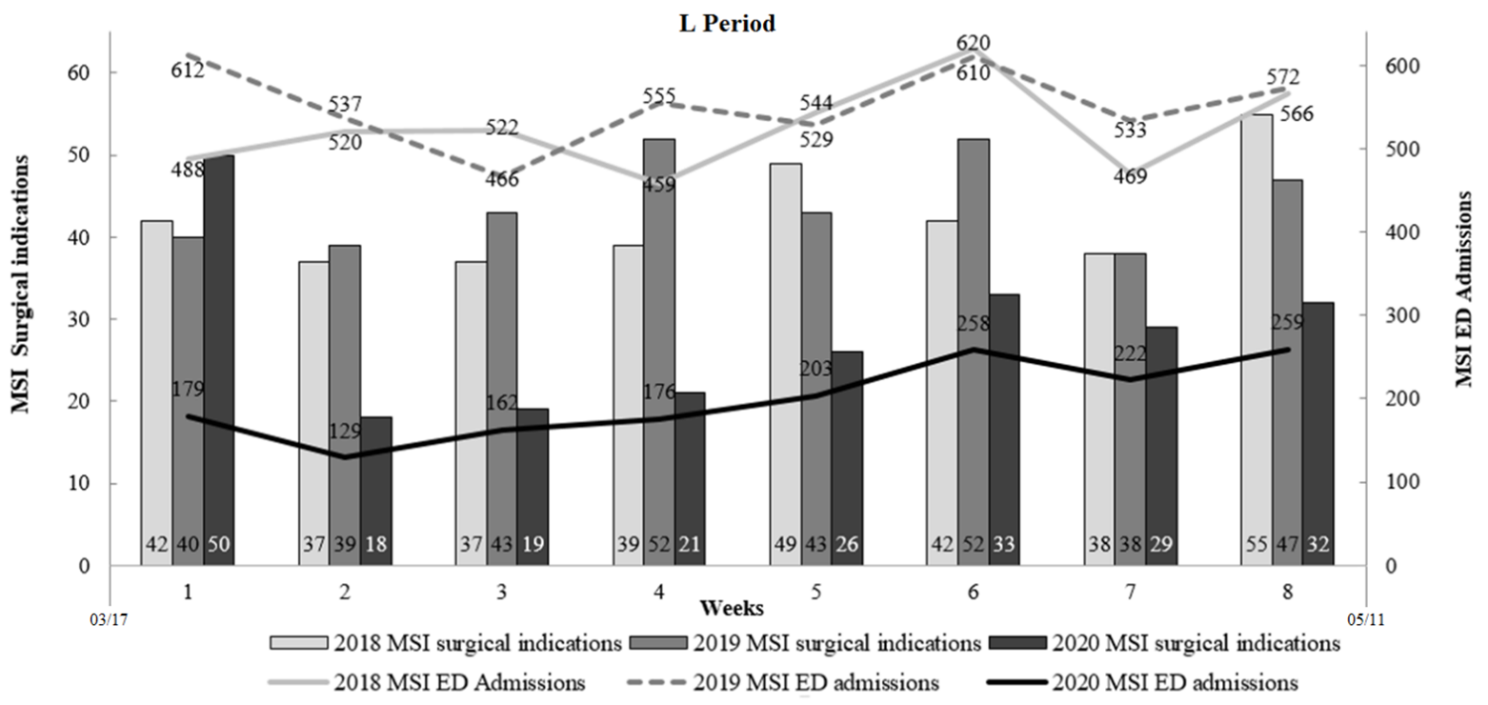


**Supplementary Figure 1:** **Change in weekly admissions for MSI in the ED and weekly surgical indications** for 2018 (03/17/18 to 05/11/18), 2019 (03/17/19 to 05/11/19), and 2020 (03/17/20 to 05/11/20) at Nantes University Hospital. MSI: Musculoskeletal injury, ED: Emergency department.

**Additional File 3:**

**Supplementary Table 2:** **Patient annual characteristics for ED admissions data and MSI surgical indications**

|  | **2018 Period** | **2019 Period** | **2020 Period** |  | |  |  |
| --- | --- | --- | --- | --- | --- | --- | --- |
|  | 56 days from 03/17/18 to 05/11/18 | 56 days from 03/17/19 to 05/11/19 | 56 days from 03/17/20 to 05/11/20 | p-value | | | |
| **ED Admission Data** | n=4188 | n=4414 | n=1588 | 2018 vs. 2020 | | 2019 vs. 2020 | 2018 vs. 2019 |
| ED MSI patients/day† | 74.8±12.9 | 78.8±12.7 | 28.4±8.2 | **<.001** | **<.001** | | 1 |
| ED MSI patients/week  Median Age †  Women | 523.5±53.4  33.7±20.6  1751 (41.8%) | 551.8±47.6  34.0±20.9  1811 (41.0%) | 198.5±46.0  43.0±22.7  641 (42.9%) | **<.001**  **<.001**  .320 | **<.001**  **<.001**  .645 | | .472  1  .462 |
| **Surgical indications Data** | n= 339 | n=354 | n=217 |  |  | |  |
| MSI surgical indication ratio | 339/4188 (8.1%) | 354/4414 (8.0%) | 217/1588 (13.7%) | **<.001** | **<.001** | | .899 |
| Surgical indications/day | 6.1±2.3 | 6.3±1.9 | 3.9±2.2 | **<.001** | **<.001** | | .987 |
| Surgical indications/week†  Median age, years†  Women | 42.4±5.8  64.0±23.4  162 (47.8%) | 44.3±6.0  60.0±24.9  160 (45.2%) | 27.1±7.4  66.0±22.7  97 (43.8%) | **.042**  .780  .477 | .056  .064  .908 | | 1  .093  .494 |

Chi² square test and one-way ANOVA test; †: Kruskal-Wallis non-parametric-test; ‡: Fisher’s exact test. ED: Emergency department; MSI: Musculoskeletal indications; ISS: Injury Severity Score. MSI: Musculoskeletal indications; ISS: Injury Severity Score.
